# A comprehensive study on decreasing the kilovoltage cone‐beam CT dose by reducing the projection number

**DOI:** 10.1120/jacmp.v11i3.3274

**Published:** 2010-05-12

**Authors:** Bo Lu, Haibin Lu, Jatinder Palta

**Affiliations:** ^1^ Department of Radiation Oncology University of Florida College of Medicine Gainesville Florida USA

**Keywords:** CBCT patient dose, image quality, registration accuracy, projection numbers, imaging reconstruction

## Abstract

The objective of this study was to evaluate the effect of kilovoltage cone‐beam computed tomography (CBCT) on registration accuracy and image qualities with a reduced number of planar projections used in volumetric imaging reconstruction. The ultimate goal is to evaluate the possibility of reducing the patient dose while maintaining registration accuracy under different projection‐number schemes for various clinical sites. An Elekta Synergy Linear accelerator with an onboard CBCT system was used in this study. The quality of the Elekta XVI cone‐beam three‐dimensional volumetric images reconstructed with a decreasing number of projections was quantitatively evaluated by a Catphan phantom. Subsequently, we tested the registration accuracy of imaging data sets on three rigid anthropomorphic phantoms and three real patient sites under the reduced projection‐number (as low as 1/6th) reconstruction of CBCT data with different rectilinear shifts and rotations. CBCT scan results of the Catphan phantom indicated the CBCT images got noisier when the number of projections was reduced, but their spatial resolution and uniformity were hardly affected. The maximum registration errors under the small amount transformation of the reference CT images were found to be within 0.7 mm translation and 0.3° rotation. However, when the projection number was lower than one‐fourth of the full set with a large amount of transformation of reference CT images, the registration could easily be trapped into local minima solutions for a nonrigid anatomy. We concluded, by using projection‐number reduction strategy under conscientious care, imaging‐guided localization procedure could achieve a lower patient dose without losing the registration accuracy for various clinical sites and situations. A faster scanning time is the main advantage compared to the mA decrease‐based, dose‐reduction method.

PACS numbers: 87.57.C‐, 87.57.cf, 87.57.cj, 87.57.cm, 87.57.cp, 87.57.N‐, 87.57.nf, 87.57.nj

## I. INTRODUCTION

Advances in three‐dimensional (3D) radiation therapy technologies have led to the safe delivery of escalated doses to tumors, improving local control and sparing dose to healthy tissues for improving quality of life.^(^ ^1^ ^)^ However, the full potential of these technologies in radiation treatment can be achieved only if the patient can be positioned accurately and reproducibly during every session of the entire course of treatment delivery.^(^ ^2^ ^)^ Mega‐voltage (MV) portal imaging has been widely implemented for the past two decades as patient treatment‐positioning tools. Due to the inherent low‐contrast and two‐dimensional nature of the projection images, the precision of MV portal imaging is limited so far as accurately defining the patient's position.^(^ ^3^ ^)^ The need for more precise patient positioning has increased the interest in developing 3D imaging techniques that can verify the patient setup immediately before and after treatment. The availability of large area flat‐panel detectors has facilitated the development of integrated cone‐beam computed tomography (CBCT) systems on linear accelerators.^(^ ^4^ ^–^ ^7^ ^)^ Until now, kilovoltage (kV) CBCT systems integrated into the gantries of linear accelerators have been widely used as an advanced image‐guided radiotherapy (IGRT) modality to acquire high‐resolution volumetric images of patients for treatment localization purposes. Using on‐line kV CBCT software and hardware, a patient's position can be determined accurately with a high degree of precision and, subsequently, setup parameters can be adjusted to deliver the intended treatment.

While the dose from a single CBCT image acquisition is small compared to the therapy dose, repeated daily image‐guidance procedures can not only lead to substantial dose to normal tissue, but also generate a high integral dose for the scanning area of the patient.^(^ ^2^ ^)^ Radiation therapy patients are already being exposed to very high and localized radiation; therefore, the additional radiation from imaging has an associated risk and should be kept low.^(^ ^8^ ^)^ Concerns about the stochastic risk of inducing cancer or genetic defects have already been accounted for in the limits on leakage from the primary therapy beam, which should not exceed 0.2% of the absorbed dose rate on the central axis at the treatment distance.^(^ ^9^ ^)^ This recommendation suggests that for a total treatment dose of 60 Gy delivered over 30 fractions, only 120 mGy or less should be contributed by MV radiation leakage. Although the energy and field of exposure differ for MV beam leakage with CBCT, making direct comparison problematic, the imaging doses are clearly not negligible. The health and safety considerations that underlie the limit on therapy‐beam background dose should, therefore, be considered as relevant for imaging exposures as well.^(^ ^9^ ^)^ How to balance imaging‐quality improvement and dose escalation risk over broad clinical scenarios is still a controversial topic in the radiation therapy community and would be beyond the scope of this article. In this study, we wanted to focus on the impact of image information resulting from dose‐reduction strategies.

In general, dose can be minimized during CBCT scan through two different means: one is by reducing the tube current; the other is by reducing the projections used in volumetric imaging reconstruction.^(^ ^9^ ^)^ The first method would result in a reduced signal‐noise ratio due to less incident photons (reduced mAs) interacting with detectors, which ultimately degrade the image quality from several perspectives (spatial resolution, soft‐tissue contrast, edge preservation, etc.). Those degrading effects have been studied by several investigators, and solutions to alleviate them have been proposed.^(^ ^10^ ^–^ ^12^ ^)^ Alternatively, the second strategy to reduce the patient's dose underlies the reduction of sampling frequency during image acquisition. The major advantage of the second method versus the first one is that it is a time saver. Faster scan speed and shorter reconstruction time could ultimately limit setup uncertainty as a result of less patient on‐couch time. Unfortunately, like the mA‐reduction method, the disadvantage of the second strategy is that image quality diminishes. More specifically, the soft‐tissue contrast degrades and streak‐shaped artifacts appear. In diagnostic imaging, there is a direct relationship between the exposure level and image quality. The demand for high‐contrast, low‐noise images pushes the exposure levels up. In IGRT, patient alignment information derived from imaging registration is the main purpose of onboard therapy imaging, which leads to a major concern with IGRT imaging – registration accuracy between reference CT and CBCT rather than general image‐quality critiques. The alignment information generated by volumetric imaging registration will eventually provide clinicians with patient setup accuracy and, subsequently, position‐adjustment guidance, if necessary. The goal of this article is to examine the effects of imaging‐registration accuracy under a dose‐decreasing strategy of reducing the projection number for different clinical sites.

A preliminary study on the strategy to decrease the radiotherapy dose by reducing the projection number was conducted by Sykes et al.^(^ ^12^ ^)^ using a head‐and‐neck phantom. Their study showed that for a specific phantom and using a reduced number of projections (one‐fifth of a full set), registration accuracy could be preserved as the on‐board imaging was reconstructed. However, several aspects of their study could potentially be revisited or further tested. First, a quantitative analysis of the degrading effects of imaging quality due to the reduced projection number was not included in their study. Since the clinician's judgment of image registration heavily depends on imaging quality, evaluating the registration accuracy with any proposed strategy is as important as assessing the degrading effects of the images, especially when automatic registration cannot provide the correct alignment information. Secondly, although head‐and‐neck phantom data demonstrate the effects of registration accuracy for a rigid body, real patient imaging can be deformed with respect to the reference CT due to setup variance, internal organ motion, and/or biological changes in the patient. It is therefore worthwhile to exam whether a proposed strategy can still be applied to nonrigid patient imaging. Furthermore, in the Sykes study, only the cross‐correlation information of the pixel gray value between image sets was used as an objective function for registration calculation, due to the availability of commercial software at that time. Today, integrated software is available for most CBCT sites, with both edge and cross‐correlation information included in the commercial software as customer options. The effects of the proposed strategy should be assessed for all of today's optional modes. Another limitation of the Sykes study is the failure to provide the results of full sets of projection‐number‐reduction schemes except for a specific scheme (one‐fifth of the projection number). We believe a more valuable study would demonstrate the registration effects with different schemes of projection‐number reduction to at least provide the researcher with opportunities to seek optimum schemes that balance image quality with patient dose. In this paper, we provide a thorough study for a dose‐decreasing method based on projection‐number reduction.

The aim of this study is to evaluate registration accuracy and image quality with a reduced number of planar projections for volumetric imaging reconstruction with an Elekta onboard CBCT. The image qualities of 3D volumetric images reconstructed with a decreasing number of projections were quantitatively evaluated using a CT phantom. Subsequently, three different sites of phantoms and patients' imaging data with different projection‐number schemes were analyzed, respectively.

## II. MATERIALS AND METHODS

### A. X‐ray volume imaging system

The X‐ray volume imaging (XVI) system (version 3.5) used in this study is an onboard kV CBCT imaging system integrated into the Elekta Synergy platform linear accelerator (Elekta Corp., Stockholm, Sweden). The XVI system consists of a kV radiation‐generation system that produces a cone‐shaped X‐ray beam, an image receptor consisting of a kV imaging panel that acquires projected planar images from the X‐ray beam, and a control system with computer workstation hardware and XVI software. The radiation generator and detector panel are both mounted on retractable arms extending from the accelerator's drum structure in an orthogonal direction to the MV system, and they share the same rotation center with a gantry‐head MV imaging‐panel rotation system. The X‐ray generator's focus to the isocenter distance is 1000 mm and the distance from the focus to the receptor is 1536 mm.

The kV generator is a 40‐kilowatt unit capable of producing single and continuous radiographic exposures of varying energy and dose. The energy range is from 40 to 150 kV. The X‐ray tube filtration is 3.5 mm of aluminum and 0.1 mm of copper. This equates to a total of approximately 6 mm of aluminum equivalence at 100 kV and 7 mm of aluminum equivalence at 120 kV. Tube current options are 25, 32, and 40 mA, while the pulse length can be set within the range of 4 to 80 ms.

The images for this study were acquired through 360° (full‐fan) or 200° (half‐fan) rotations. The pulse rate for full‐fan and half‐fan were 2.7 Hz and 5.4 Hz, respectively. The fast gantry speed was 60 seconds. The regular scanning speed was about 120 seconds. Pixel size was $0.8\;{\text{mm}}^{2}$. Each projection image was sampled with 512×512 pixels. The maximum image volume was defined by the size of the collimator, and the maximum correspondent field of view was 26 cm.

XVI software was running on a clinical workstation with 2.4 GHz CPU and 2 GB of memory. The reconstruction algorithm provided by the XVI is Feldkamp's back‐projection algorithm^(^ ^13^ ^)^ considering flex‐map correction in rotation orbits.^(^ ^14^ ^)^

### B. Phantoms and patient selection

#### B.1 Catphan phantom

Image quality drastically impacts the clinician's judgment of registration accuracy when auto‐registration cannot provide correct registration results, especially for nonrigid body registration.^(^ ^9^ ^)^ Typically, quantitative image‐quality tests are performed by analyzing a specially designed CT phantom scan. For instance, Catphan phantom (model CTP 503, The Phantom Laboratory, Salem, NY) has been recommended by the Elekta XVI system as the acceptance test phantom. Reconstructed Catphan phantom images with varying projection‐number schemes were accessed in our study through three major criteria specified in the acceptance test guide. The Catphan phantom is cylindrical and has multiple layers embedded with different shapes and materials of inserts. It could be used to examine CT number uniformity, spatial resolution, low‐contrast resolution, and geometric accuracy, among other factors.

#### B.2 RANDO phantom

Three body parts of a male RANDO phantom (The Phantom Laboratory, Salem, NY) were selected as rigid imaging objects for our registration‐accuracy study. We refer to them as the head‐and‐neck area, thoracic area, and pelvic area as R‐H&N, R‐Thoracic, and R‐Pelvis, respectively. The phantom was constructed with a natural human skeleton cast inside of soft tissue‐simulating material. In the thoracic area, lungs are molded to fit the contours of a natural rib cage. The air space of the head, neck and stem bronchi are duplicated. The phantom is sliced at 2.5 cm intervals. The whole grid patterns can be drilled into the sliced sections to enable the insertion of dosimeters. Two tissue‐simulating materials are used to construct the RANDO Phantom: the RANDO soft tissue material and the RADNO lung material. Both of these are designed to have the same absorption as human tissue at the normal radiotherapy and radiology exposure levels. The RANDO's similarities to a real human structure make it an attractive and widely used imaging and dosimetry substitute for simulation among the radiology community.

#### B.3 Patient sites

Three patients' image sets at different sites were used for our registration study. They were a brain‐cancer site, a lung‐cancer site, and a prostate‐cancer site, which are referred to as P‐Brain, P‐Lung and P‐Prostate, respectively. The reference CT images were taken a week before treatments for planning purpose, whereas CBCT images were taken immediately prior to patient treatment to register with reference CT images for localization purpose. Unlike rigid phantom imaging, the real patients imaging data could be deformed between reference CT and CBCT image sets. For example, patient's prostate could be stretched or squeezed by its surrounding tissues and organs. (Different amount of urine inside bladder and/or different amount of gas inside rectum between the moments of acquiring reference CT and CBCT can cause different shapes of the prostate in these two image sets.) Similarly, chest cage could be enlarged or shrunk depending on the phase of breathing while acquiring images. Since the registration solution of nonrigid imaging is much easier to trap into a local minima solution, assessing the robustness of the registration algorithm with nonrigid patient data would make the study more valuable and thorough.

### C. Acquisition and reconstruction of CBCT images

The positioning procedure used with the Catphan phantom followed the acceptance test guide of the XVI. The RANDO phantom and patients were placed centrally in the field of imaging view prior to the scan. All CBCT images of phantoms and patients were acquired by the standard technique recommended by XVI protocol systems. The technique parameters including beam energy, tube current, duration per pulse, projected image number, scan time, gantry rotation angle and collimator type, are listed in Table 1. Since the recommended technique could generate near‐optimum image quality for the current system, those 3D images reconstructed with full sets of projected planar images were treated as standard images for the registration study and referred to as “XVI‐full.” The near‐optimum image qualities for each site were achieved by choosing high tube currents and high energy beams so as to minimize noises and any reconstruction artifacts due to high‐density materials inside the phantoms or patients. A deliberate, slow gantry speed also benefitted the image quality since it allowed enough projected images to be acquired during the slow gantry rotation in order to reconstruct images with minimal artifacts and structural noise generated from the high‐contrast edge. The term “optimum image quality” in our study refers to the best image quality achievable by the current system with minimal noise and artifacts, and thus minimal impact on the accuracy of the 3D image registration.

**Table 1:** Technical parameter lists of scan for phantoms and patients.

	*Catphan*	*R‐H&N*	*R‐Thoracic*	*R‐Pelvis*	*P‐Brain*	*P‐Lung*	*P‐Prostate*
Energy (kVp)	120	100	120	120	100	120	120
Tube Current (mA)	30	10	40	40	10	40	40
Duration per Pulse (millisecond)	40	10	25	25	10	25	40
Projected Image Number	652	362	652	652	362	652	652
Scan Time (minutes)	2.0	2.0	2.0	2.0	2.0	2.0	2.0
Start Angle–end Angle (degree)	183–180	260–100	183–180	183–180	260–100	183–180	183–180
Collimator Type	S20	S20	M20	M20	S20	M20	M10

Abbreviations: kVp=kilovolta peak; mA=milli‐Amper R‐H&N=head‐and‐neck‐area RANDO phantom; R‐Thoracic=thoracic‐area RANDO phantom; R‐Pelvis=pelvic‐area RANDO phantom; P‐Brain=brain site; P‐Lung=lung site; P‐Prostate=prostate site; S20=small size collimator with 20 cm width; M10=medium size collimator with 10 cm width; M20=medium size collimator with 20 cm width.

All 3D images were reconstructed using $1\;{\text{mm}}^{3}$ voxels. The registration outcome of the XVI full scan with the corresponding “reference planning CT scan” dataset was applied as the baseline to evaluate the registration accuracy of images reconstructed with fewer projections. In order to simulate a series of lower doses and faster scans, the reconstruction schemes with fewer projections consisted of half, one‐third, one‐fourth, one‐fifth, and one‐sixth of the full projections, referred to as XVI‐1/2, XVI‐1/3, XVI‐1/4, XVI‐1/5, and XVI‐1/6, respectively. The image quality degrading effects along with the fewer numbers of projections were analyzed. As the projection number became less than one‐sixth of the full set, unacceptable image quality became an obstacle to most clinicians who could not provide sound judgments about registration results due to the low visibility in the soft tissue contrast. Therefore, even though reconstructed images with projections less than one‐sixth of full set would potentially sustain the edge information, registration test with a projection number less than one‐sixth of full set have been excluded from our study.

3D imaging reconstructions were performed with XVI software. The schemes with fewer projection numbers were achieved by manually deactivating the undesirable projection frames in the XVI database. For instance, XVI‐1/n was reconstructed only with frames whose index number could be divisible by the number *n*, and all other frames would not be used for reconstruction at all. Projection‐number schemes used for patients and phantoms are listed in Table 2. It should be noted that this was a feasibility study and XVI‐1/n does not mean that the scan speed was increased by *n* times, but rather that it was limited by the gantry‐speed limitations permitted by the International Electrotechnical Commission (IEC) and achievable tube firing frequency. The validity of simulating a low‐dose fast scan will be discussed.

**Table 2:** Projection number of different schemes for phantoms and patients used in study.

	*Catphan*	*R‐H&N*	*R‐Thoracic*	*R‐Pelvis*	*P‐Brain*	*P‐Lung*	*P‐Prostate*
XVI‐full	668	362	652	652	362	652	652
XVI‐1/2	334	181	326	326	181	326	326
XVI‐1/3	223	121	217	217	121	217	217
XVI‐1/4	167	91	163	163	91	163	163
XVI‐1/5	134	72	130	130	72	130	130
XVI‐1/6	111	60	109	109	60	109	109

Abbreviations: XVI‐ful=a full set of projected planar images, XVI−1/2=half a set of projected planar images, etc.; R‐H&N=head‐and‐neck‐area RANDO phantom; R‐Thoracic=thoracic‐area RANDO phantom; R‐Pelvis=pelvic‐area RANDO phantom; P‐Brain=brain site; P‐Lung=lung site; P‐Prostate=prostate site.

### D. Imaging quality check with a reduced projection number

The influence of a reduced number of projections on image quality was evaluated using the Catphan phantom. The evaluated items included 3D spatial resolution, 3D low‐contrast visibility, and 3D uniformity. The “CAT‐Image Quality” preset parameters (referred to in Table 1) were used to acquire the volumetric images. A 360° rotation scan was performed and a total of 668 projections were registered in the XVI database. The reconstructed 3D images with all projection number schemes were tested separately. A detailed description and criteria of those tests follow.

#### D.1 3D spatial resolution

**Figure 1:**
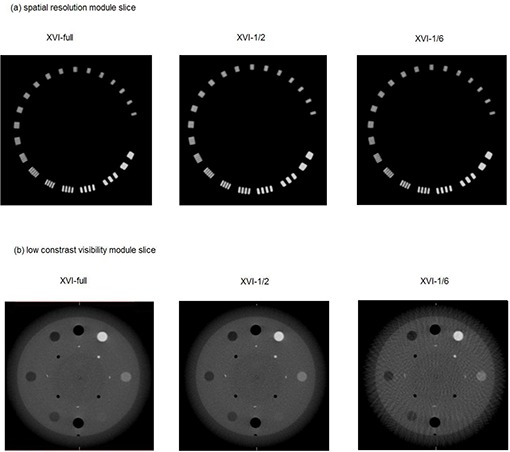
A spatial‐resolution module slice (a) of a Catphan phantom image reconstructed with XVI‐full, XVI‐1/2, and XVI‐1/6 schemes; a low‐contrast visibility module slice (b) of a Catphan phantom image reconstructed with XVI‐full, XVI‐1/2, and XVI‐1/6 schemes.

We located the spatial resolution module slice of the reconstructed CBCT image, then magnified the image until the module filled the transverse view, and subsequently determined the highest numbered line pairs visible by adjusting the brightness and contrast to achieve the best view for the line pairs. The specification was greater or equal to seven line pairs per centimeter. Figure 1(a) shows the reconstructed images with XVI‐full, XVI‐1/2, and XVI‐1/6 schemes at the spatial resolution module slice, respectively.

#### D.2 3D low‐contrast visibility

Figure 1(b) shows the contrast resolution module slice reconstructed with XVI‐full, XVI‐1/2, and XVI‐1/6 schemes for the 3D low‐contrast visibility test. This test measures the mean pixel values of the polystyrene insert (7 o'clock, Fig. 1(b)) and the LDPE insert (9 o'clock, Fig. 1(b)), as well as the standard deviation of their pixel values. The low‐contrast visibility value was defined mathematically as Eq. (1), where *SD* represents the standard deviation of the pixel values, *Mean* represents the mean of the pixel value; and the subscripts (*polystyrene* and *LDPE*) represent two different materials (polystyrene and low‐density polystyrene), respectively. If the calculated value of Eq. (1) was large, it meant the difference of imaging noises between these two materials overwhelmed the difference of true imaging signals between them, indicating poor low contrast visibility. The reverse also is true. The XVI specification for the low‐contrast visibility value was lower than 2%.

(1) $$low-contrast\;visiblity=\frac{5.5}{\left\{\left[Mean_{polystyrene}-Mean_{LDPE}\right]/{\;}_{\left[\frac{SD_{polystyrene}-SD_{LDPE}}{2}\right]}\right\}}$$

#### D.3 3D uniformity

The uniformity module slice of the Catphan phantom was used as the image for the 3D uniformity test. Four random locations in the uniformity module were chosen to measure the mean values of the pixels covered by a $1\times 1\;{\text{cm}}^{2}$ probe window. Equation (2) was used to compute the maximum percentage difference between any two of the mean pixel values, where *max(mean)* and *min(mean)* are the largest and smallest mean pixel values among the four locations, respectively. The specification was less than or equal to 2%.

(2) $$uniformity=\frac{Max(mean)-Min(mean)}{Max(mean)}$$

### E. Registration analysis

The reference CT images of the phantoms and patients were acquired by a Philips multi‐slice high‐speed CT scanner (Amsterdam, The Netherlands). The placement of the phantoms allowed the scan origin to be near the isocenter used in XVI imaging, but no effort was taken to ensure that the center was identical or that the phantom was rotationally aligned in the CT scanner with respect to the XVI scans. Real patient setup, however, strictly followed department setup procedures to avoid any possible rotation variance between the reference CT scan and XVI scan for practical clinical purposes.

Automatic 3D registration of the reference CT and the XVI scans was performed using XVI software. Two automatic registration modes were used for our study. The “bone mode” of the automatic registration uses a chamfer matching algorithm originally described by Borgefors.^(^ ^15^ ^)^ The chamfer algorithm performs registration by matching the edge information of two image sets. It is relatively insensitive to image noise and mild streak artifacts, and also has a quick computing time. It performed poorly with lower‐contrast subjects only. Comparisons of the quality of registration with the bone mode with other techniques have been provided by several investigators.^(^ ^16^ ^,^ ^17^ ^)^ The “grey value mode” performs the auto‐registration by matching voxel grey‐scale intensity values throughout the entire interested image volume, which is well known as the “cross‐correlation” technique.^(^ ^18^ ^)^ In addition to the edge information, soft tissue information has also been included in the grey‐value algorithm and would potentially average out the registration error caused by anatomy variance between the two image sets. In terms of calculation time, the grey‐value mode is much worse than the bone mode in general. In our study, we had tried the bone mode for all patient and phantom images, and the grey‐value mode only for patient images of pelvis and lung sites.

The performance of the automatic registration was checked by repeatedly registering the reference CT planning scan with the XVI scan for each projection number scheme. We initially registered reference CT imaging with XVI‐full imaging and considered the registration results as “Gold Standards”. The recorded translation and rotation values were then treated as baseline and were subtracted from the values of the registration results of other schemes to acquire the registration error values for all schemes accordingly. To simulate setup error in terms of translation and rotation, we manually entered the translation value in the x‐, y‐, and z‐axes and rotation angle in the x, y, and z directions to achieve the desirable transformed reference CT datasets. Three independent translations with values of 2 mm, 5 mm and 20 mm along each axis of x, y, and z, and two independent rotations with values of 3° and 10° around each axis of x, y, and z, were applied to the reference CT scan. The transformed reference CT images were then registered with XVI images reconstructed with different numbers of projections to test the registration accuracy. Additionally, two combinations of translation and rotation were applied to the reference CT planning scan for all three axes, to access the registration capability with various scheme reconstructions for more realistic reference CT transforms as well. To demonstrate that registration was reproducible, each registration procedure was performed 10 times repeatedly, and the mean and standard deviation values in terms of translation vector error and maximum rotation angle error of all directions were recorded.

## III. RESULTS

### A. Reconstructed images

**Figure 2:**
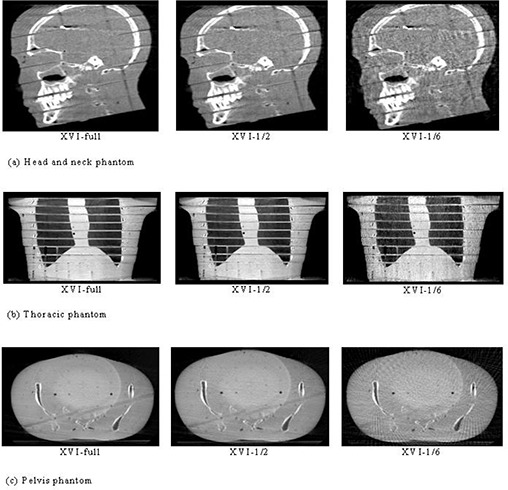
Head‐and‐neck phantom (a) with images of a RANDO phantom including XVI‐full, XVI‐1/2, and XVI‐1/6 reconstruction image slices in the sagittal view; thoracic phantom (b) with images of a RANDO phantom including XVI‐full, XVI‐1/2, and XVI‐1/6 reconstruction image slices in the coronal view; pelvis phantom (c) with images of the pelvis area of a RANDO phantom including XVI‐full, XVI‐1/2, and XVI‐1/6 reconstruction image slices in the axial view.

**Figure 3:**
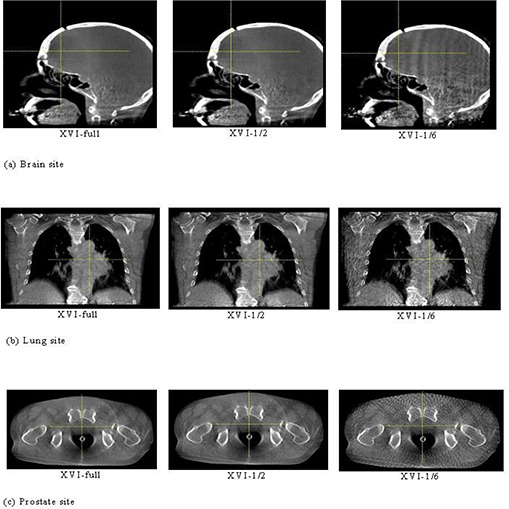
Brain site (a): the patient images of the brain site including XVI‐full, XVI‐1/2, and XVI‐1/6 reconstruction image slices in the sagittal view; lung site (b): patient images of the lung site including XVI‐full, XVI‐1/2, and XVI‐1/6 reconstruction image slices in the coronal view; prostate site (c): patient images of the prostate site including XVI‐full, XVI‐1/2, and XVI‐1/6 reconstruction image slices in the axial view.

The XVI reconstruction time of patients and phantoms with different projection‐number schemes are listed in Table 3. Obviously, fewer projection numbers resulted in shorter computing times. XVI‐1/6 reconstruction procedures lasted an average of 4.8 seconds over all patients and phantoms, which is about 1/6th of the average time spent for all XVI‐full reconstructions. The representative trans‐axial, sagittal and coronal slices of the XVI‐full, XVI‐1/2, and XVI‐1/6 schemes for three RANDO phantom parts and three different patient sites are shown in Figs. 2 and 3, respectively. As predicted, detailed contrast of soft tissue in XVI imaging could not be visualized as well as with reference CT imaging, even with full‐projection reconstruction. XVI‐full showed some low‐frequency noise artifacts that created nonuniformity on the grey level in the tissue‐equivalent material and some streak‐shaped artifacts originating from high‐contrast edges (like the couch top). It was noticed that XVI‐1/2 images were slightly noisier than XVI‐full; however, streak artifacts were not more severe than XVI‐full, while edge information between the anatomy interfaces was well kept. XVI‐1/6 images presented significant streak artifacts with the appearance of an interference pattern due to the under‐sampling effect of the reconstruction. Reduced visibility in the overall soft tissue contrast was also observed in the XVI‐1/6 images. However, even in the XVI‐1/6 images, the gross bony anatomy and air cavity were geometrically undistorted and a surprising level of fine edge details still remained, whereas the presence of degrading effects of soft tissue contrast escalated.

**Table 3:** XVI computing time of reconstructions for patients and phantoms with different projection‐number schemes.

	*Catphan*	*R‐H&N*	*R‐Thoracic*	*R‐Pelvis*	*P‐Brain*	*P‐Lung*	*P‐Prostate*
XVI‐full (seconds)	31.2	23.7	30.0	34.2	33.1	30.3	28.9
XVI‐1/2 (seconds)	18.5	13.6	17.8	18.9	17.9	17.3	16.3
XVI‐1/3 (seconds)	15.3	10.5	13.2	15.0	14.1	14.1	13.6
XVI‐1/4 (seconds)	8.8	5.4	8.1	9.6	9.0	8.2	7.2
XVI‐1/5 (seconds)	6.2	3.0	5.7	8.0	6.1	5.9	5.2
XVI‐1/6 (seconds)	5.3	2.4	4.9	6.0	5.3	5.1	4.8

Abbreviations: XVI‐ful=a full set of projected planar images, XVI−1/2=half a set of projected planar images, etc.; R‐H&N=head‐and‐neck‐area RANDO phantom; R‐Thoracic=thoracic‐area RANDO phantom; R‐Pelvis=pelvic‐area RANDO phantom; P‐Brain=brain site; P‐Lung=lung site; P‐Prostate=prostate site.

### B. Imaging quality check

The image quality results were provided quantitatively as follows:

#### B.1 3D spatial resolution check

Figure 1(a) presents the image slice of the spatial‐resolution module of the Catphan phantom with XVI‐full, XVI‐1/2, and XVI‐1/6 reconstruction schemes separately. The highest number of visible line pairs of XVI‐1/6 is seven. Increases in projection number led to better visibility of line pairs, especially for XVI‐full and XVI‐1/2 for which line‐pair 8 could easily be visualized. In summary, all tests satisfied the criteria (visibility of line‐pair 7) provided by the XVI acceptance manual.

#### B.2 3D low‐contrast visibility check

**Figure 4:**
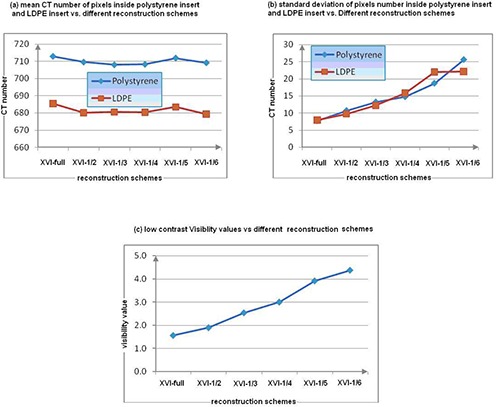
Results of a low‐contrast visibility check with different reconstruction schemes.

Figure 1(b) presents the 3D low‐contrast visibility module slice of the Catphan phantom with XVI‐full, XVI‐1/2, and XVI‐1/6 reconstruction schemes separately. Clearly, the imaging interference from noises became more severe as the number of projections used for 3D reconstruction decreased. Figures 4(a) and 4(b) show the mean CT number and standard deviation of pixels inside the two inserts regions (described in Methods and Materials section) versus different reconstruction schemes, respectively. The results show that mean pixel value curves were almost flat, whereas the standard deviation increased as the number of projections decreased, indicating that increased noise due to under‐sampling does significantly influence detailed contrast visibility. Figure 4(c) summarizes the calculated values of low‐contrast visibility by Eq. (1) versus the different reconstruction schemes. The XVI specification for the low‐contrast visibility value was less than 2%. Only XVI‐full and XVI‐1/2 met the specification; others failed. More specifically for a clinic scenario, the degradation due to under‐sampling effect would be the appearance of soft tissue contrast lost in CBCT images. For instance, muscle tissues could be clearly visualized in XVI‐full image set of the prostate patient (Fig. 3(c)), whereas the appearance of muscle tissues in XVI‐1/6 images had been degraded (merged by surrounding tissues due to high noises).

#### B.3 3D uniformity check

**Figure 5:**
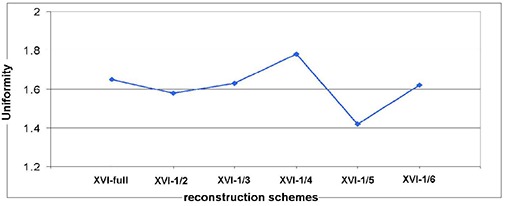
Results of a uniformity check with different reconstruction schemes.

All tests met the criteria. The results are presented in Fig. 5. No signs indicate that uniformity worsened with fewer projection numbers.

### C. Registration

**Figure 6:**
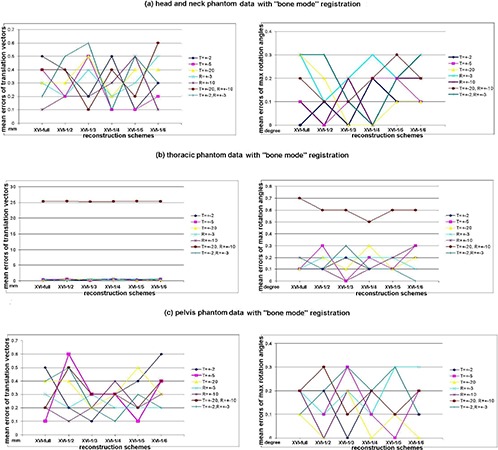
The scatter charts of mean registration errors of the translation vector and mean registration errors of maximum rotation angles of all directions versus reconstruction schemes with different translation and rotation scales for phantoms with “bone mode” registration algorithm: (a) head‐and‐neck phantom data with the “bone mode” registration; (b) thoracic phantom data with the “bone mode” registration; (c) pelvis phantom data with the “bone mode” registration. Note: all mean registration error values of the translation vector were rounded to one‐tenth of a millimeter; all mean registration error values of maximum rotation angles were rounded to one‐tenth of a degree.

**Figure 7:**
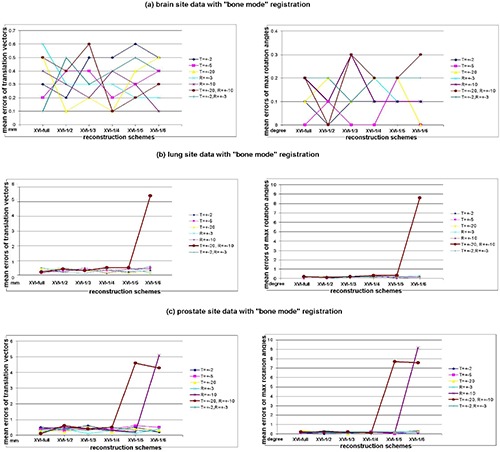
The scatter charts of mean registration errors of the translation vector and mean registration errors of maximum rotation angles of all directions versus reconstruction schemes with different translation and rotation scales for all patient sites with “bone mode” registration algorithm: (a) brain site data with the “bone mode” registration; (b) lung site data with the “bone mode” registration; (c) prostate site data with the “bone mode” registration. Note: all mean registration error values of the translation vector were rounded to one‐tenth of a millimeter; all mean registration error values of maximum rotation angles were rounded to one‐tenth of a degree.

**Figure 8:**
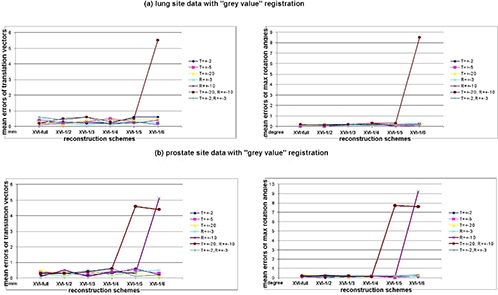
The scatter charts of mean registration errors of the translation vector and mean registration errors of maximum rotation angles of all directions versus reconstruction schemes with different translation and rotation scales for non‐rigid patient sites with “grey value” registration algorithm: (a) lung site data with the “grey value” registration; (b) prostate site data with the “grey value” registration. Note: all mean registration error values of the translation vector were rounded to one‐tenth of a millimeter; all mean registration error values of maximum rotation angles were rounded to one‐tenth of a degree.

The mean registration errors of 10 repeated measurements in terms of translation vector error and maximum rotation‐angle error versus different reconstruction schemes are presented as scatter charts in Fig. 6 for Phantoms with “bone mode” registration algorithm, Fig. 7 for all patient sites with “bone mode” registration algorithm and Fig. 8 for nonrigid patient sites with “grey mode” registration algorithm. Each chart located at left side of each subfigure shows the result data of translation vector error; the one at right side shows the result data of maximum rotation‐angle error. Since each subfigure had the same structures, we only describe them in general. In the left‐side chart of each subfigure, the x‐axis represented the different schemes of XVI reconstruction; the y‐axis represented the mean registration error of 10 repeated measurements for the translation vector only; and the seven series labels with different shapes and colors represent the seven different translation and rotation combinations applied to the reference CT images. To facilitate the visibility of overlapped error points across different series, the color lines between adjacent points of same series have been added to help the reader distinguish those error points among different series. These combination series consisted of 2 mm shifts along the x‐, y‐, and z‐axes called “T=2 mm,” 5 mm shifts in the x‐, y‐, and z‐axes called “T=5 mm,” 20 mm shifts in the x‐, y‐, and z‐axes called “T=20 mm,” 3° rotations in x, y, and z directions called “R=3°,” 10° rotations in x, y, and z directions called “R=10°” 2 mm shifts along the x, y, and z axes plus 3° rotations in the x, y, and z directions called “T=2 mm, R=3°,” and 20 mm shifts along the x‐, y‐, and z‐axes plus 10° rotations in the x, y, and z directions called “T=20 mm, R=10°”. In each right‐side chart of each subfigure, the y‐axis represented means of the maximum angle error over all directions of 10 repeated measurements. (The other labels had the same meaning as the right‐side chart and will not be repeated here.) Standard deviations of the registration error over 10 repeated measurements were 0.1 mm or less in translation and 0.10 or less in rotation across the board. The superimposed standard deviation bars were excluded from the charts to avoid the visibility interference of mean values. The mean registration time by bone mode was 4.2 seconds and the standard deviation was 1.3 seconds; the mean registration time by grey‐value mode was 15.6 seconds and the standard deviation was 3.2 seconds.

## IV. DISCUSSION

The results indicate that, for image sets of rigid body (e.g. brain phantom, pelvis phantom and brain site), when treatment volume can be determined by bone–tissue and/or tissue–air interface, without clinician interference, auto‐registration results using reduced‐projection‐number (up to one‐sixth of projections) reconstruction can still provide accurate enough alignment information with no obvious trends of error escalation while projection number decreased. Error points were randomly scattered in charts (shown in Fig. 6(a), Fig 6(c) and Fig 7(a)) with maximum errors of 0.7 mm in translation and 0.3° in rotation – except of the thoracic phantom, for which a large misalignment (10° rotations and/or 20 mm translation shown in Fig. 6(b)) was applied. For nonrigid anatomy sites like the lung and prostate, however, auto‐registration with too few (one‐fifth or one‐sixth) projection‐number reconstructions could lead to greater alignment errors (shown in Fig. 7(b), Fig. 7(c), Fig. 8(a), Fig. 8(b)), unless manual pre‐alignment was applied. Meanwhile, the overall reproducibility of registration results was shown to be excellent (less than 0.1 mm; 0.1° standard deviation), whereas other geometric uncertainties that exist in the radiotherapy treatment process are much more significant.

Three major image quality parameters with different scheme reconstructions are shown in Figs. 2–4. As demonstrated, high‐contrast details and spatial resolution were well preserved even with the lowest projection‐number reconstruction (XVI‐1/6) tested, whereas low‐dose fast scans may not be sufficient to visualize low‐contrast soft tissue with an ultra‐low projection‐number reconstruction due to increased noise and streak artifacts resulting from under‐sampling effects. It could be concluded that for rigid‐bony anatomy sites, like the brain, an ultra‐low projection‐number‐reduction strategy may still be a trouble‐free solution to tremendously reducing the patient dose, even if soft tissue contrast visibility was completely lost. Whereas, for non‐rigid soft‐tissue anatomy sites, like the prostate and lung, reconstruction with too few projections could trap the registration results into local minima, making the clinician's interference for registration unavoidable. Thus, it is much more sensible to optimize soft tissue visibility for rigid sites versus nonrigid sites. It is conceivable that imaging processing techniques that could reduce noise and enhance soft tissue contrast would be preferred when soft tissue contrast becomes prominent for imaging registration. It was also noticed that Figs. 2(c) and 3(c) showed some ring artifacts caused by imperfect CBCT detector calibration in current version of XVI software. They are common artifacts for all the ELEKTA machines. In general, we assumed subtle ring artifacts should not influence registration accuracy since registration results were dominated by bony structure. However, to validate this assumption, a sophisticated comparison between registration outcomes with ring artifact removed and without removal is needed. Due to limited accessibility to the imaging data, such analysis is not feasible for this study.

As mentioned, a local minima trapping situation occurred with the ultra‐low projection reconstruction (XVI‐1/4) for nonrigid sites (lung and prostate) due to the combined effects of increased noises, nonrigid anatomy, and large misalignments. For these two sites, as the 10° rotation and ultra‐low reconstruction scheme were applied simultaneously, large translation and rotation errors led to the registration results failing regular clinical registration criteria (2 mm in translation and 3° in rotation), which indicates that reconstruction with too few projection numbers (XVI‐1/4 or worse) will be the “scare factor” for some non‐rigid anatomy sites. Initial human interference is imperative to overcome the local minima trapping effect. Another local minima trapping situation occurred while we were performing rigid thoracic phantom registrations with a setup simulation that had a large initial misalignment. As “R=10°” or “T=20 mm, R=10°” were applied to the reference CT, the registration results were mismatched by one complete vertebra superiorly or inferiorly due to the similarity of adjacent vertebra and ribs in the thoracic region. Like the nonrigid cases, this mismatching could be overcome by initial manual alignment followed by the auto‐registration procedure. For a real patient's lung, auto‐registration after applying a large misalignment performed fairly well simply because a real patient has more irregular internal structures than a phantom structure (compare Figs. 2(b) and 3(b)). Therefore, the registration results are less likely to fall into the local minima and there is more time to seek a global minima solution. This mismatching occurred for all schemes from XVI‐full to XVI‐1/6 so that the projection number was irrelevant.

In our study, bone mode and grey‐value mode did not present any significant differences for all schemes of reconstruction in terms of registration accuracy. However, the computing speed was the key point distinguishing these two methods. On average, the bone mode ran almost 4 times faster than the grey‐value mode. Thus, the bone mode would be recommended to save on procedure time for applicable cases.

The reconstruction time for XVI‐1/6 is 5.2 seconds (on average) using the XVI software. Considering that the average XVI‐full reconstruction time was 30 seconds, we could save roughly 25 seconds for reconstruction, which is helpful but not significant. However, considering clinical settings, image reconstruction is typically concurrent with image acquisition and the reconstruction time is masked; therefore, reconstruction time‐saving is not an important factor. The real time‐saving factor could be generated by the fact of fewer projections needed during scanning, as mentioned previously. However, even though XVI‐1/6 reconstruction only needed 1/6th of the full‐set projection number, it does not appear that that the potential scan time for the XVI‐1/6th scheme could be escalated by 6 times. For example, a XVI‐full scan of 652 projections needs an average of 2 minutes for the pelvis, whereas a 110‐projection scan could not be accomplished in 20 seconds due to the current limitations on the maximum gantry speed, the highest tube firing frequency, and patient safety concerns. Since the maximum gantry speed is limited to no greater than 60/s (in order to comply with IEC regulations),^(^ ^19^ ^)^ less than a 1 minute scanning time is impossible according to current safety regulations. Therefore the scanning time could be reduced from current 2 minutes to 1 minute. Since the system is designed for automatic registration integrated with software for XVI acquisition and reconstruction, then it should be possible to perform the complete image‐guided process of image acquisition, reconstruction, registration and correction of patient positioning within 3 minutes instead of the usual 5 minutes. This would make on‐line image guidance a practical and realistic procedure

Previous discussions indicate that an XVI‐full scan is not necessary when auto‐registration could provide accurate alignment information. For all the cases in our study, it appears that XVI‐1/4 would allow a good balance between dose reduction and accurate enough registration. The study by Song et al.^(^ ^20^ ^)^ elucidates that for the average person, the brain, lung and prostate doses to the patient's surface (i.e., the highest dose to the patient) per XVI‐full scan are 0.22 cGy, 4.6 cGy, and 2.4 cGy, respectively. Intuitively, a XVI‐1/4 scan would give one‐fourth of the dose of an XVI‐full scan to the patient's surface for any site. More specifically, 0.055 cGy would be the skin dose from a XVI‐1/4 brain site scan, and for the lung site and prostate site they would be 1.15 cGy and 0.6 cGy, respectively. If we assume that the average prescription dose for IMRT or 3DCT is 200 cGy, and we perform a cone‐beam scan at each fraction, the percentage dose from XVI to MV would be 0.02%, 0.6%, and 0.3%. Adopting 0.2% of a MV dose as the dose limit recommended by Murphy et al.,^(^ ^9^ ^)^ the XVI dose to the brain site by a XVI‐1/4 scan would satisfy this dose limit; dose from XVI‐1/4 scan for the other two sites would near the limit. In hypo‐fractionation stereotactic body radiotherapy cases, the patient's average MV dose is 1000 cGy, so the CBCT dose for all sites would be well below the limit. However, since no safe radiation level exists, the ALARA (”as low as reasonably achievable”) standard for non‐target doses for concomitant exposures is always recommended by the radiology community. The radiation carcinogenesis risk for patients undergoing radiation therapy is a subject of much discussion in the scientific community.^(^ ^21^ ^)^ Nevertheless, the techniques presented here show that there is a potential to significantly reduce the XVI dose without sacrificing the precision of the image‐guided procedure.

Before we conclude our paper, we would like to point out that even though our study was limited to the Elekta CBCT systems due to hardware and software availability, the presented strategy and evaluation methodology could be applied in other commercial KV‐IGRT systems or even for MV‐IGRT systems as well. Analysis of projection‐number reduction strategy for other commercial IGRT systems would be a worthwhile research to complement this study in future.

## V. CONCLUSIONS

This study has demonstrated that image guidance for radiotherapy in a rigid anatomy can be achieved using as little as 1/6th of the regular scan dose with an Elekta Synergy XVI system through a projection‐number‐reduction strategy. However, reconstruction with a projection number lower than 1/4th of the full set potentially traps the registration into local minima for nonrigid anatomies, and initial clinician interference may be needed to achieve better registration accuracy. A faster scanning time is the main advantage compared to the mA‐based dose‐reduction method. Ultimately, the projection‐number‐reduction method would potentially reduce the patient‐on‐couch time and alleviate setup uncertainties due to patient movement throughout the IGRT setup procedure.
